# The relationship between study findings and publication outcome in anesthesia research following implementation of mandatory trial registration: A systematic review of publication bias

**DOI:** 10.1371/journal.pone.0282839

**Published:** 2023-05-26

**Authors:** Simon W. Chong, Georgina Imberger, Amalia Karahalios, Andrew Wang, Millicent Burggraf, Maleck Louis, Grace M. Liskaser, Anthony Bianco, Philip J. Peyton

**Affiliations:** 1 Department of Critical Care, The University of Melbourne, Melbourne, Australia; 2 Department of Anaesthesia, Pain and Perioperative Medicine, Western Health, Melbourne, Australia; 3 Centre for Epidemiology and Biostatistics, Melbourne School of Population and Global Health, The University of Melbourne, Melbourne, Australia; 4 Department of Anaesthesia, Austin Health, Melbourne, Australia; 5 Department of Surgery, Austin Hospital, Melbourne, Australia; UT Southwestern: The University of Texas Southwestern Medical Center, UNITED STATES

## Abstract

Previously, we reviewed 1052 randomized-controlled trial abstracts presented at the American Society of Anesthesiologists annual meetings from 2001–2004. We found significant positive publication bias in the period examined, with the odds ratio for abstracts with positive results proceeding to journal publication over those with null results being 2.01 [95% confidence interval: 1.52, 2.66; P < 0.001]. Mandatory trial registration was introduced in 2005 as a required standard for publication. We sought to examine whether mandatory trial registration has decreased publication bias in the anesthesia and perioperative medicine literature. We reviewed all abstracts from the 2010–2016 American Society of Anesthesiologists meetings that reported on randomized-controlled trials in humans. We scored the result of each abstract as positive or null according to a priori definitions. We systematically searched for any subsequent publication of the studies and calculated the odds ratio for journal publication, comparing positive vs null studies. We compared the odds ratio from the 2010–2016 abstracts (post-mandatory trial registration) with the odds ratio from the 2001–2004 abstracts (pre-mandatory trial registration) as a ratio of odds ratios. We defined a 33% decrease in the odds ratio as significant, corresponding to a new odds ratio of 1.33. We reviewed 9789 abstracts; 1049 met inclusion criteria as randomized-controlled trials, with 542 (51.7%) of the abstracts going on to publication. The odds ratio for abstracts with positive results proceeding to journal publication was 1.28 [95% CI: 0.97, 1.67; P = 0.076]. With adjustment for sample size and abstract quality, the difference in publication rate between positive and null abstracts was statistically significant (odds ratio 1.34; 95% CI: 1.02, 1.76; P = 0.037). The ratio of odds ratios, comparing the odds ratio from the 2010–2016 abstracts (post-mandatory trial registration) to the odds ratio from the 2001–2004 abstracts (pre-mandatory trial registration), was 0.63 (95% CI: 0.43, 0.93); P = 0.021). We present the first study in the anesthesia and perioperative medicine literature that examines and compares publication bias over two discrete periods of time, prior to and after the implementation of mandatory trial registration. Our results suggest that the amount of publication bias has decreased markedly following implementation of mandatory trial registration. However, some positive publication bias in the anesthesia and perioperative medicine literature remains.

## Introduction

Systematic reviews with meta-analyses are often used to guide evidence-based clinical practice. There are many sources of error in the conclusions of meta-analyses. Positive publication bias, being the selective submission and acceptance for publication of studies with positive over those with null results [1], is an important source of systematic error that is difficult to quantify and adjust for, leading to unreliable conclusions and the potential to misinform clinical practice.

To address publication bias, and also selective outcome reporting, the International Committee of Medical Journal Editors (ICMJE) in 2005 introduced mandatory prospective trial registration of clinical trials as a pre-requisite for acceptance for publication [2]. Unfortunately, a large number of journals were still publishing randomized-controlled trials (RCTs) without prospective registration almost a decade after the introduction of mandatory trial registration [3]. Previous studies have demonstrated a low proportion of prospective trial registration in recent times across multiple fields of medicine [4–6].

Previously, we conducted a review of 1052 RCT abstracts presented at the American Society of Anesthesiologists (ASA) annual meetings between 2001 and 2004 [7]. We found an odds ratio (OR) for abstracts with positive results proceeding to journal publication over those with null results of 2.01 [95% confidence interval (CI): 1.52, 2.66; P < 0.001], demonstrating the presence of significant positive publication bias in the anesthesia and perioperative medicine literature over the period examined.

In the current study, we sought to examine whether mandatory prospective trial registration has decreased publication bias in the anesthesia and perioperative medicine literature, despite evidence suggestive of low proportions of trial registration. We measured the publication bias present in the anesthesia and perioperative medicine literature 5 years after the introduction of mandatory prospective trial registration, by undertaking a review of trial findings and subsequent publication outcome in peer-reviewed journals of all RCTs presented and published as conference abstracts at the ASA annual meetings over the period 2010–2016. After calculating our estimate of publication bias for this post-mandatory trial registration sample, we then tested for a significant difference to our previous pre-mandatory trial registration sample from 2001–2004 [7].

## Methods

This study was prospectively registered and protocol uploaded on The University of Melbourne Minerva database on the 21 February 2018 (http://hdl.handle.net/11343/198340). Ethics board approval was not required for this retrospective observational study. The applicable PRISMA guidelines [8] were adhered to.

**Fig 1:**
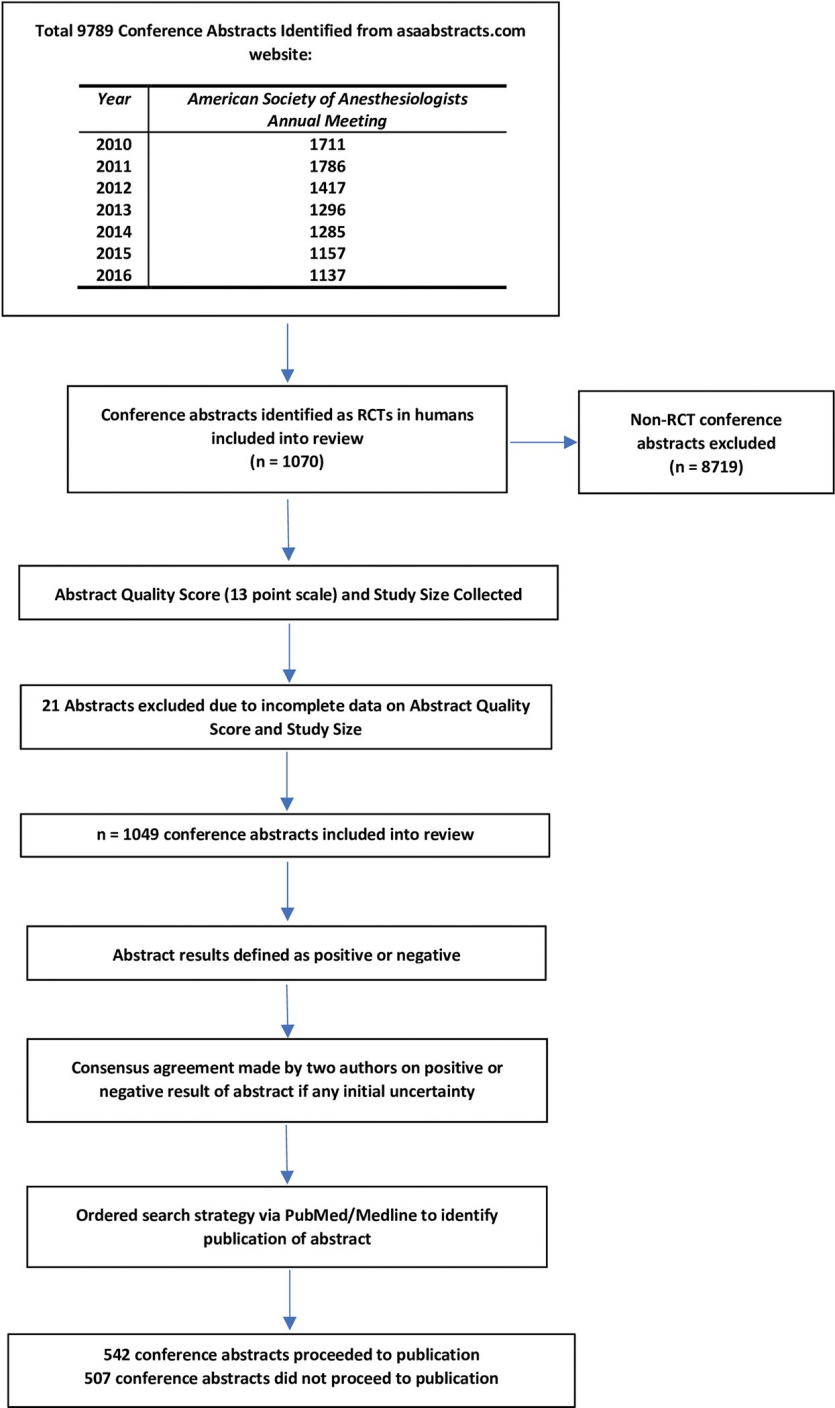
Flow diagram of review.

Utilising the ASA Abstracts website (www.asaabstracts.com), all listed abstracts describing RCTs in humans and presented at the ASA Annual Meetings between 2010–2016 were identified (Fig 1) and included in a Microsoft Excel 2019 database (Microsoft Corporation, Redmond, WA, USA). This timeline was chosen as it corresponds to a period five years after the implementation of mandatory trial registration. It also allowed five years to have elapsed for any subsequent publication of studies in the peer reviewed literature to occur. Findings from our previous review [7] demonstrated that at 5 years post abstract presentation, 94% of the positive abstracts and 92% of the null abstracts that eventually went on to publication, had been published.

Initially, data collection was performed for all abstracts from the 2010–2013 ASA Meetings. However, a lower than expected number of conference abstracts met our inclusion criteria as RCTs in humans. In order to reach the estimated required sample size, additional data collection through the review of all abstracts from the 2014–2016 ASA Meetings was then conducted.

The methods used from our previous review [7] were used for this study. A trial was considered to be an RCT if the individuals in the trial were prospectively assigned to one of two or more alternative healthcare interventions using a random method of allocation [9]. Positive studies were defined as studies showing a statistically significant treatment effect in the direction of the experimental treatment for the primary outcome compared to the control treatment, in the conference abstract. If there was no clear definition of the primary outcome, the first reported outcome was considered as the primary outcome. If the stated objective was to show treatment equivalence or non-inferiority, then studies showing no difference in outcome between treatment groups were counted as positive. Null studies were defined as studies that failed to show a statistically significant treatment effect in the direction of the experimental treatment for the primary outcome compared to the control treatment, or failed to show equivalence or non-inferiority if that was the stated aim, in the conference abstract. If there was any uncertainty over the result of the abstract, two authors reviewed the abstract and made a consensus decision guided by the above definitions.

We searched PubMed and Medline to identify any subsequent publication of the study. The search strategy included seven separate searches in the order of the first author’s name, the second author’s name, the last author’s name, the first author’s name AND keywords, the second author’s name AND keywords, the last author’s name AND keywords, and keywords alone.

A comparison of the sample size in the conference abstract to any subsequent journal publication was performed to elicit if full recruitment had been completed by the time of conference abstract presentation. If there was a discrepancy between the two values, the study was classified as incomplete at time of conference presentation, unless explicitly stated in the abstract as having completed full recruitment.

We utilised a scoring system consisting of 13 variables (S1 Table) adapted from a checklist created by Hopewell et al. [10], based on existing reporting standards [11], in order to assess the quality of the conference abstracts. Each variable was scored as “one” if present, or “zero” if absent, to provide a total score out of 13. Time to publication from conference abstract presentation date of those studies that proceeded to journal publication was also calculated. This was determined by calculating the number of months from the abstract presentation at the ASA conference (October of the relevant year) through to the month of the journal issue in which the associated publication appeared.

### Sample size

Using the database from our previous review [7], a simulation was run which determined that a sample size of 1052 abstracts would be required for the new sample to detect an estimated 33% decrease in OR, with 80% power.

### Statistical analysis

Statistical analyses were performed using Microsoft Excel 2019 (Microsoft Corporation, Redmond, WA, USA) and Stata version 16.1 (StataCorp. 2019. Stata Statistical Software: Release 16. College Station, TX: StataCorp LLC).

To estimate the association between study outcome (positive/null) and whether the abstract was subsequently published (yes/no), we fitted univariable and multivariable logistic regression models to estimate the OR. The multivariable logistic regression model included sample size and abstract quality score. The primary endpoint was then a comparison between the unadjusted ORs for study outcome (positive/null and publication [yes/no]) for the period 2001–2004 (pre-mandatory trial registration) [7] versus the period 2010–2016 (post-mandatory trial registration), by fitting an interaction term in the model (i.e. a ratio of ORs). We defined a 33% decrease from this value in OR to be of significant importance, corresponding to a new OR of 1.33. The threshold of a one-third reduction in the OR was achievable with our projected sample size, and we deemed this to represent a substantial change in publication bias.

Next, we fitted separate univariable and multivariable linear regression models to estimate the association between publication (yes/no) and outcome (positive/null), with abstract quality scores and sample size on the log scale. The multivariable models included sample size and abstract score. As a post-hoc analysis, we fitted a poisson regression model with robust standard error (Appendix 2) to estimate the risk ratio (RR) for the association between study outcome (positive/null) and whether the abstract was subsequently published (yes/no).

Time to publication (for abstracts that were published) was compared between positive and null studies by fitting Cox regression models with years since publication as the time metric. The multivariable model included abstract score and sample size.

Finally, we assessed how the number of RCT abstracts presented at the ASA meetings has changed from 2010 to 2016 by presenting a scatter plot of number of abstracts by year and fitting a linear regression model.

## Results

We reviewed 9789 abstracts presented at the ASA Meetings between 2010–2016. 1049 abstracts met the inclusion criteria as RCTs in humans. From the 1049 included abstracts, 542 (51.7%) subsequently went on to publication (Table 1).

**Table 1:** Data on publication outcomes (published in peer-reviewed journals or unpublished) and study findings (positive or null) of randomized-controlled trials presented as abstracts at the American Society of Anesthesiologists annual meetings in 2010–2016.

	All	Published		Not published	
		Positive	Null	Positive	Null
Abstracts eligible (n)	1049	402	140	351	156
Study size*	60 [37, 100]	62 [40, 110]	75 [46, 148]	50 [31, 80]	50 [36, 79]
Abstract quality score*	8 [7, 9]	8 [7, 9]	8 [7, 9]	8 [7, 9]	8 [7, 9]
Not completed at conference abstract n (%) **		283 (70.4)	103 (73.6)		
Study size difference (journal vs conference abstract)*		0 [0, 4]	0 [0, 5]		
Time to publication (months)*		16 [7, 29]	18 [8, 27]		

* Summary statistics presented as median (25th, 75th percentiles).

**For studies proceeding to journal publication, the number (%) of studies that were not completed at the time of conference presentation is shown.

Using the univariate model, without any adjustments for sample size and quality of the abstracts, the OR for abstracts with positive results proceeding to journal publication compared to those with null results was 1.28 [95% confidence interval (CI): 0.97, 1.67; P = 0.076] (Table 2) (the equivalent RR is presented in S2 Table). The ratio of ORs comparing this OR from the 2010–2016 period to the unadjusted OR from the 2001–2004 period was 0.63 (95% CI: 0.43, 0.93); P = 0.021).

**Table 2:** Analysis of the relationships between publication outcomes (published in peer-reviewed journals or unpublished) and study findings (positive or null) of randomized-controlled trials presented as abstracts at the American Society of Anesthesiologists annual meetings 2001–2004 versus 2010–2016.

	Pre-Mandatory Trial Registration Period (2001–2004)																						
	**Not published** **(n = 488)**			**Published** **(n = 564)**				**Univariable model**								**Multivariable model** *							
**Conclusion**	n	%		n		%		Odds ratio		95% CI—lower limit		95% CI—upper limit		p-value		Odds ratio		95% CI—lower limit		95% CI—upper limit		p-value	
**Null**	166	34.0		115		20.4																	
**Positive**	322	66.0		449		79.6		2.01		1.52		2.66		<0.001		2.02		1.53		2.67		<0.001	
	**Post-Mandatory Trial Registration Period (2010–2016)**																						
	**Not published** **(n = 507)**				**Published** **(n = 542)**				**Univariable model**								**Multivariable model** *						
**Conclusion**	n		%		n		%		Odds ratio		95% CI—lower limit		95% CI—upper limit		p-value		Odds ratio		95% CI—lower limit		95% CI—upper limit		p-value
**Null**	156		30.8		140		25.8																
**Positive**	351		69.2		402		74.2		1.28		0.97		1.67		0.076		1.34		1.02		1.76		0.037

* Multivariable model adjusted for sample size and abstract quality score.

• **The ratio of ORs for the univariable model comparing the OR from the 2010–2016 period to the OR from the 2001–2004 period was 0.63 (95% CI: 0.43, 093); P = 0.021.**

• **The ratio of ORs for the multivariable model comparing the OR from the 2010–2016 period to the OR from the 2001–2004 period was 0.67 (95% CI: 0.45, 0.99); P = 0.043.**

With adjustment for sample size and abstract quality, the OR for publication outcome related to a positive study result was slightly higher and statistically significant (OR 1.34; 95% CI: 1.02 to 1.76; P = 0.037) (Table 2).

When we compared the abstract quality directly, there was no difference in mean abstract quality scores [SD] between studies proceeding to journal publication and studies which did not (7.9[1.20] vs. 7.9[1.14]; mean difference from multivariable model = 0.05 (95% CI: -0.09, 0.19); P = 0.499) (Table 3). Similarly, mean abstract quality scores [SD] did not differ between positive and null studies (7.9[1.14] vs 8.0[1.24]; mean difference from multivariable model = -0.13 (95% CI: -0.29, 0.02); P = 0.096).

**Table 3:** Analysis of the relationships between publication outcomes (published in peer-reviewed journals or unpublished) and study findings (positive or null), sample size, and abstract quality score, of randomized-controlled trials presented as abstracts at the American Society of Anesthesiologists annual meetings 2010–2016.

Outcome = Abstract score (out of 13)																		
			Univariable model								Multivariable model*							
**Published**	N	Mean (SD)	Mean difference		95% CI—lower limit		95% CI—upper limit		p-value		Mean difference		95% CI—lower limit		95% CI—upper limit		p-value	
No	507	7.9 (1.14)	Ref								Ref							
Yes	542	7.9 (1.20)	0.04		-0.10		0.18		0.559		0.05		-0.09		0.19		0.499	
**Conclusion**																		
Null	296	8.0 (1.24)	Ref								Ref							
Positive	753	7.9 (1.14)	-0.13		-0.29		0.03		0.103		-0.13		-0.29		0.02		0.096	
Outcome = sample size																		
				Univariable model								Multivariable model*						
	N	Median (25th, 75th percentiles)		Geometric mean ratio		95% CI—lower limit		95% CI—upper limit		p-value		Geometric mean ratio		95% CI—lower limit		95% CI—upper limit		p-value
**Published**																		
No	507	50.0 (32.00, 80.00)		Ref								Ref						
Yes	542	65.0 (40.00, 120.00)		1.37		1.23		1.52		<0.001		1.38		1.24		1.53		<0.001
**Conclusion**																		
Null	296	60.0 (39.5, 107.0)		Ref								Ref						
Positive	753	60.0 (36.0, 99.0)		0.89		0.79		1.00		0.052		0.87		0.77		0.98		0.021

*Multivariable model adjusted for publication (no/yes) and conclusion (null/positive).

The median (25^th^, 75^th^ percentiles) sample size for abstracts proceeding to journal publication was 65 (40, 120) compared to 50 (32, 80) for conference abstracts that did not (Table 3). The estimated relative change in geometric mean sample size from the multivariable model was 1.38 (95% CI: 1.24, 1.53; P < 0.001) comparing published abstracts to those that weren’t published. The median (25^th^, 75^th^ percentiles) sample size for abstracts with positive results was 60 (36, 99) compared to 60 (39.5, 107) for those with null results. The estimated relative change in geometric mean sample size from the multivariable model was 0.87 (95% CI: 0.77, 0.98; P = 0.021) comparing positive results to those with a null result.

**Fig 2:**
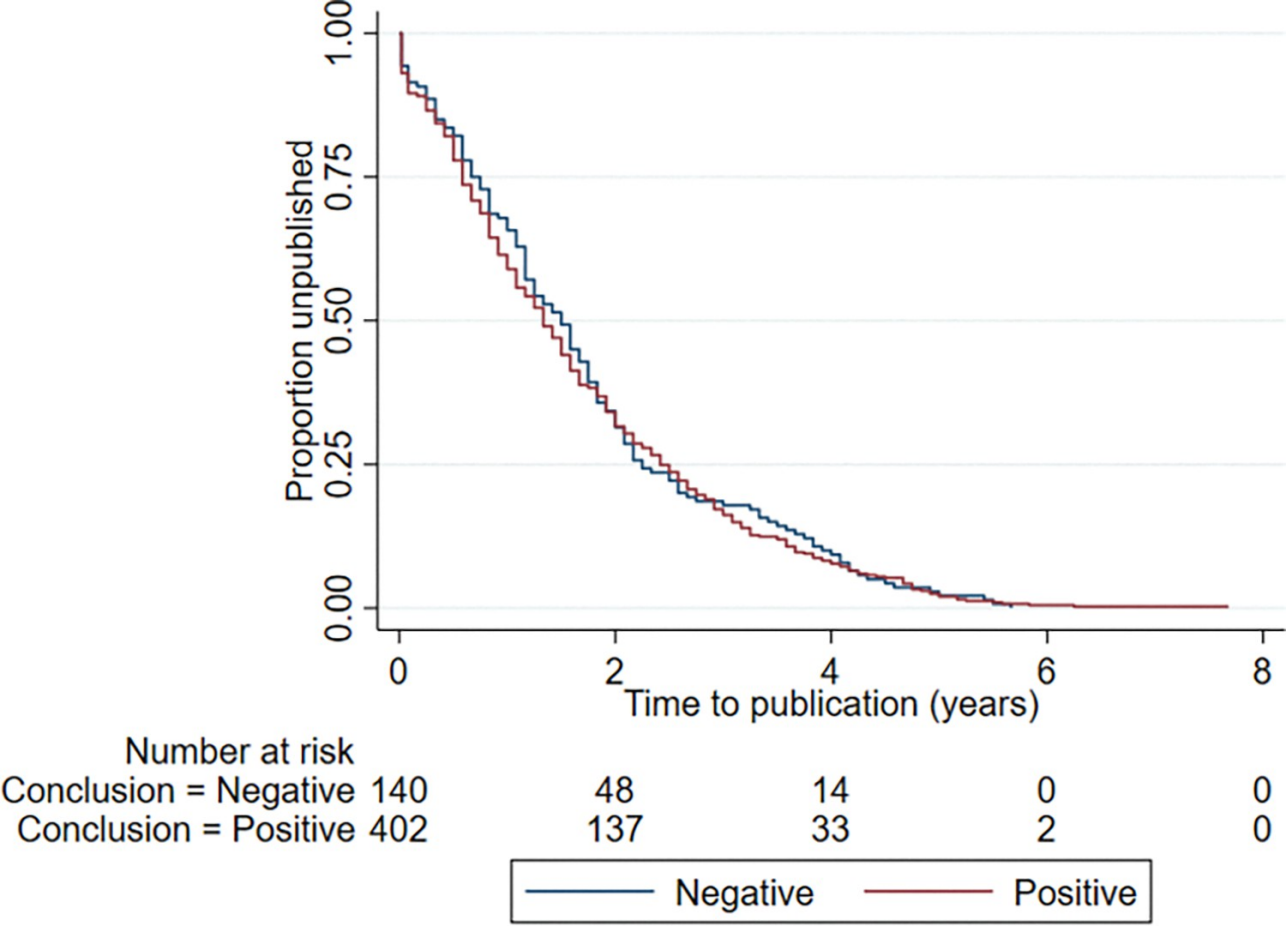
Time to publication in years for the positive and null studies that proceeded to publication. The number at risk (remaining unpublished studies in each group) is shown.

The time to publication of abstracts ranged from -15 months (published 15 months prior to presentation at the ASA Meeting) to 92 months (Fig 2). When comparing positive and null studies, there was no difference between the median time in years (25^th^, 75^th^ percentiles) to publication, (positive studies: 1.3 (0.6–2.4) years and null studies: 1.5 (0.7–2.3) years) respectively. The hazard ratio comparing time to publication for positive vs null studies was 1.05 (95% CI: 0.87, 1.28; P = 0.597).

From the 542 studies that went on to publication, 283 (70.4%) of the positive studies and 103 (73.6%) of the null studies were classified on review as not completed at the conference abstract phase. This was due to the sample size of the conference abstract differing to the final sample size in the corresponding journal publication. The median (25^th^, 75^th^ percentiles) difference in sample size was minor for both positive 0 (0, 4) and null 0 (0, 5) studies.

**Fig 3:**
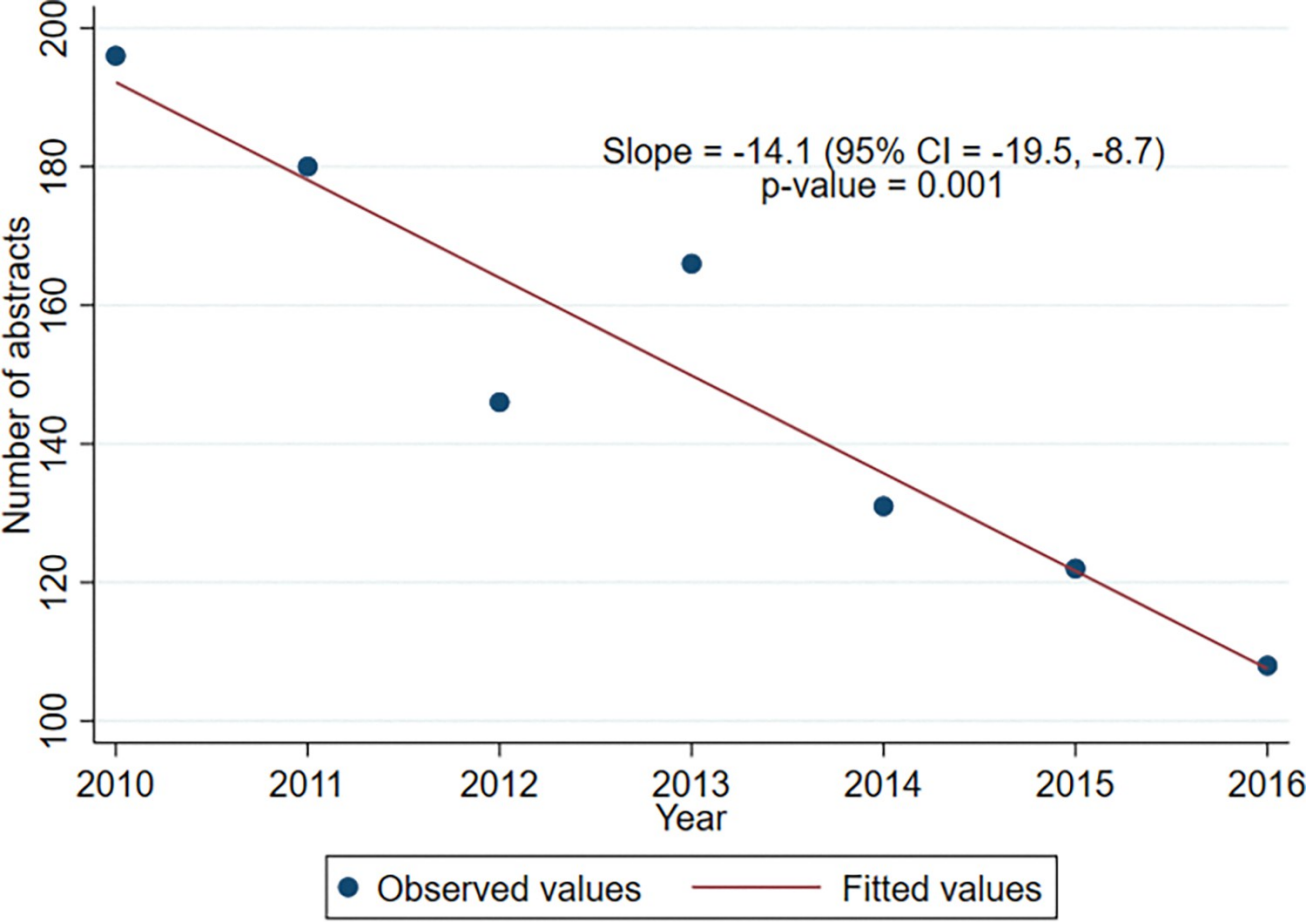
Number of randomized-controlled trials presented at the American Society of Anesthesiologists annual meetings from 2010–2016.

Fig 3 shows the number of RCT abstracts presented each year at the ASA meetings, which has decreased from 2010 to 2016, with a gradient of -14.1 RCTs per subsequent year (95% CI: -19.5, -8.7; P = 0.001).

## Discussion

From a review of 1049 RCT abstracts presented at the largest annual scientific conference in anesthesia and perioperative medicine, greater than five years after the introduction of mandatory prospective trial registration, we found that studies with a positive result were 1.28 times more likely to be published than those with null results (OR [95% CI] 1.28 [0.97, 1.67]). This contrasts significantly (P = 0.021) to our previous findings from a review of 1052 RCT abstracts examining a period of time prior to the introduction of mandatory prospective trial registration, when publication bias was greater (OR for studies with positive results proceeding to publication: 2.01; 95% CI: 1.52, 2.66) [7].

The OR [95% CI] of 1.28 [0.97, 1.67] was modified to 1.34 [1.02, 1.76] when adjustment was made for sample size and abstract quality score. Consequently, our results suggest the presence of ongoing significant, albeit markedly less, positive publication bias in the anesthesia and perioperative medicine literature over the period 2010–2016 when compared to the period 2001–2004.

Publication bias occurs in all fields of health research [12–15]. However, the ability to measure publication bias in a prospective manner is limited by the availability and access to unpublished manuscripts. In the setting of anesthesia, De Oliveira et al. [16] found that 72% of publications in four high impact journals had positive conclusions, compared with 53% in lower impact journals, and that the odds ratio [95% CI] for publication in a high impact anesthesia journal if the article had positive results was 2.28 [1.76, 3.01]. This was an indirect method of measurement, focusing on potential bias in the submission and review process of journals. Sources of publication bias, however, can occur earlier in the research process, including the failure to complete a study or to submit for publication. Understanding and quantifying all the contributors to publication bias is necessary to mitigate the associated effects.

Consequently, due to the presence of the carefully maintained ASA abstracts website, we were able to utilise a full source of studies presented at the ASA annual meetings, regardless of their future publication status, for our investigation. This provided us with the ability to effectively quantify publication bias in a selected large sample of anesthesia and perioperative medicine literature. Our methodology of defining the population on the basis of conference abstracts instead of published papers, thus provided a window into an earlier phase of the research process, and provided a discriminator that was otherwise not available from the consideration of published work only.

The data in Table 2 show that there was a large majority of positive studies at both the pre- and post-publication stages of the research process, and this was present during both time windows that we compared. While it may be expected that the process of hypothesis driven trials might naturally lead to a predominance of positive findings (e.g. investigators’ clinical experience leading more often to investigation of correct hypotheses), nevertheless we showed that the publication phase of the process increased this predominance, without evidence of justification in the form of demonstrably better study size or quality. However, the magnitude of this increase was substantially smaller in the later window, indicating a decrease in publication bias after the introduction of mandatory trial registration.

Our findings did, however, pose the question whether it was the introduction of mandatory prospective trial registration that has resulted in the reduction of publication bias. Rates of compliance with this policy by authors and journals are central to this question. Multiple studies, in various settings, have measured persistently low rates of trial registration in the years after it became mandatory [4–6,17].

In the setting of anesthesia, De Oliveira et al’s review of RCTs published in 2013 in the five highest impact factor anesthesia journals [17] found that 64% of the trials were not prospectively registered. In a similar time period, Jones et al found only 12% of the RCTs adequately prospectively registered in the top six general anesthesia journals by impact factor [4]. This was similar to our own findings from a review of RCT abstracts presented at the ASA meetings from 2010–2016 [18], from which only 21% had undergone prospective trial registration.

Consequently, our findings of decreased publication bias cannot be explained by high rates of compliance with mandatory prospective trial registration. It may be that the introduction of mandatory prospective trial registration, and other changes aimed at improving the quality of RCTs, could have provided an effective deterrent from performing small-sized, underpowered or low quality RCTs [19]. If low-quality trials are more likely to be published based on being positive than a negative high-quality trial, then a reduction of low-quality trials due to mandatory prospective trial registration would decrease publication bias, independent of the actual rates of prospective registration.

There is empirical support for this argument. In the study by Jones et al., as a post hoc secondary outcome, the authors noted a “large decline in the absolute number of RCTs being reported in the anesthesia literature” [4]. Whilst this finding could be related to increasing difficulty or costs related to performing RCTs, shifting of publications to smaller journals, or favouring of less onerous observational study designs [4], it could also be the result of researchers collaborating on larger, well designed multi-centre RCTs, albeit with fewer trials in total number.

Data from our study supports this argument as well, with a post-hoc analysis revealing an average decline of -14.1 (95% CI: -19.5, -8.7; P = 0.001) RCTs being presented over each successive year at the ASA meetings from 2010–2016 (Fig 3).

Our study has several limitations. This trend of decreased RCTs forced a change to our study protocol. We initially planned to review 4 years of RCT abstracts from the 2010–2013 ASA meetings (similar to our previous study [7]). However, we needed a further 3 years to reach our sample size. Importantly, there was still a minimum period of 5 years in which publication could occur. Our previous review [7] demonstrated that almost all studies had been published at 5 years post abstract presentation (94% of positive abstracts and 92% of negative abstracts). So this extension was unlikely to compromise the validity of our findings.

As with our previous review [7], there was no evidence from the abstract scores to support a systemic difference in the quality of abstracts presented at the 2010–2016 ASA meetings, when comparing studies with positive versus null results. In addition, the abstract scores were similar between the different periods of time examined (2001–2004 vs 2010–2016). However, widespread differences in protocol, trial design and study power influencing publication cannot be ruled out, given the varied assortment of RCTs presented at the ASA meetings.

When examining the influence of sample size on publication outcome, abstracts not proceeding to publication had a 23% smaller median sample size. Reassuringly there was no difference in sample size between positive and null studies. In addition, in similar fashion to our previous study, we found no difference in time to publication between positive and null studies, thereby making it unlikely that there was any significant bias arising from differences in the stages of the research and publication process between positive or null studies.

Our findings offer some encouragement that publication bias has reduced in the setting of anesthesia and perioperative medicine. Publication bias is still present but it has improved since the introduction of mandatory prospective trial registration. As compliance with mandatory prospective trial registration remains low, it is not clear what has caused publication bias to improve.

### Conclusions

We compared publication bias over two discrete periods of time, prior to and after the implementation of mandatory prospective trial registration, in the setting of anesthesia and perioperative medicine. Our results suggest that the rate of publication of studies with positive results over those with null results, and thus publication bias, has decreased markedly in the period following the implementation of mandatory prospective trial registration. However, there is still an ongoing presence of positive publication bias and the maintenance and introduction of new effective strategies for minimising this remains important. In particular, increased compliance with mandatory prospective trial registration will lead to further improvements.

## Supporting information

S1 Table Abstract quality scoring system. (DOCX)

S2 Table Analysis of the relationships between publication outcomes (published in peer-reviewed journals or unpublished) and study findings (positive or null) of randomized-controlled trials presented as abstracts at the American Society of Anesthesiologists annual meetings 2001–2004 versus 2010–2016, utilising risk ratio (RR). (DOCX)

S1 File PRISMA checklist. (DOCX)
