# Supplementary material for: The relationship between study findings and publication outcome in anesthesia research following implementation of mandatory trial registration: A systematic review of publication bias
Source: PLoS One. 2023 May 26;18(5):e0282839. doi: 10.1371/journal.pone.0282839 (PMC10218755; doi:10.1371/journal.pone.0282839)
Supplement: S1 Table — (DOCX) [file pone.0282839.s001.docx]

# **S1 Table. Abstract Quality Scoring System.**

| Scoring of Abstract Quality (13 Criteria) |
| --- |
| 1. Study objectives described |
| 1. Date of trial given |
| 1. Blinding discussed |
| 1. Inclusion / exclusion criteria described |
| 1. Experimental intervention described |
| 1. Comparator intervention described |
| 1. Baseline characteristics of the patient groups described |
| 1. Primary outcome measure described |
| 1. Number of patients analysed stated |
| 1. Intention to treat principle described |
| 1. Important adverse effects described |
| 1. Results described |
| 1. Primary conclusions described |
