# Supplementary material for: The relationship between study findings and publication outcome in anesthesia research following implementation of mandatory trial registration: A systematic review of publication bias
Source: PLoS One. 2023 May 26;18(5):e0282839. doi: 10.1371/journal.pone.0282839 (PMC10218755; doi:10.1371/journal.pone.0282839)
Supplement: S2 Table — (DOCX) [file pone.0282839.s002.docx]

# **S2 Table. Analysis of the relationships between publication outcomes (published in peer-reviewed journals or unpublished) and study findings (positive or null) of randomized-controlled trials presented as abstracts at the American Society of Anesthesiologists annual meetings 2001-2004 versus 2010-2016, utilising risk ratio (RR).**

|  | **Post-Mandatory Trial Registration Period (2010-2016)** | | | | | | | | | | | |
| --- | --- | --- | --- | --- | --- | --- | --- | --- | --- | --- | --- | --- |
|  | **Not published**  **(n = 507)** | | **Published**  **(n = 542)** | | **Univariable model** | | | | **Multivariable model*** | | | |
| **Conclusion** | n | % | n | % | Risk ratio | 95% CI - lower limit | 95% CI - upper limit | p-value | Risk ratio | 95% CI - lower limit | 95% CI - upper limit | p-value |
| **Null** | 156 | 30.8 | 140 | 25.8 |  |  |  |  |  |  |  |  |
| **Positive** | 351 | 69.2 | 402 | 74.2 | 1.13 | 0.98 | 1.30 | 0.084 | 1.14 | 0.99 | 1.31 | 0.067 |
|  | * Multivariable model adjusted for sample size and abstract quality score | | | | | | | | | | | |
